# Bioluminescence imaging visualizes osteopontin-induced neurogenesis and neuroblast migration in the mouse brain after stroke

**DOI:** 10.1186/s13287-018-0927-9

**Published:** 2018-07-04

**Authors:** Rebecca Rogall, Monika Rabenstein, Sabine Vay, Annika Bach, Anton Pikhovych, Johannes Baermann, Mathias Hoehn, Sébastien Couillard-Despres, Gereon Rudolf Fink, Michael Schroeter, Maria Adele Rueger

**Affiliations:** 1 Department of Neurology, University Hospital of Cologne, Kerpener Strasse 62, 50924 Cologne, Germany; 2 Max Planck Institute for Metabolism Research, Cologne, Germany; 3 Institute of Experimental Neuroregeneration, Spinal Cord Injury and Tissue Regeneration Center Salzburg (SCI-TReCS), Paracelsus Medical University, Salzburg, Austria; 4 Cognitive Neuroscience Section, Institute of Neuroscience and Medicine (INM-3), Research Centre Juelich, Juelich, Germany

**Keywords:** Doublecortin, Microglia, Neural progenitor cells, Photothrombosis

## Abstract

**Background:**

Osteopontin (OPN), an acidic phosphoglycoprotein, is upregulated in the brain after cerebral ischemia. We previously reported that OPN supports migration, survival, and proliferation of neural stem cells (NSC) in primary cell culture, as well as their differentiation into neurons. We here analyzed the effects of OPN on neuroblasts in vivo in the context of cerebral ischemia.

**Methods:**

Transgenic mice expressing luciferase under the control of the neuroblast-specific doublecortin (DCX)-promoter, allowing visualization of neuroblasts in vivo using bioluminescence imaging (BLI), were injected with OPN intracerebroventricularly while control mice were injected with vehicle buffer. To assess the effects of OPN after ischemia, additional mice were subjected to photothrombosis and injected with either OPN or vehicle.

**Results:**

OPN enhanced the migration of neuroblasts both in the healthy brain and after ischemia, as quantified by BLI in vivo. Moreover, the number of neural progenitors was increased following OPN treatment, with the maximum effect on the second day after OPN injection into the healthy brain, and 14 days after OPN injection following ischemia. After ischemia, OPN quantitatively promoted the endogenous, ischemia-induced neuroblast expansion, and additionally recruited progenitors from the contralateral hemisphere.

**Conclusions:**

Our results strongly suggest that OPN constitutes a promising substance for the targeted activation of neurogenesis in ischemic stroke.

## Background

Osteopontin (OPN) is an acidic phosphoglycoprotein of about 41,500 Da that was first described as being secreted by transformed cells isolated from bone [1, 2]. Nowadays, OPN is known to be expressed in response to injury, stress, and inflammation in many types of cells such as osteoclasts, hepatocytes, vascular smooth muscle cells, epithelial cells, endothelial cells, macrophages, and activated T cells [2–7]. As a ubiquitously expressed protein, OPN is involved in homeostasis, angiogenesis, wound healing, and also immune responses [8–11]. In the brain, OPN is expressed constitutively and upregulated after cerebral ischemia [12, 13]. In the subacute stage of cerebral ischemia, OPN is upregulated in microglia and macrophages within the infarct core and in peri-infarct regions [11, 14, 15]. After ischemic stroke, OPN shows two distinct beneficial effects: a direct neuroprotective effect on neurons [16, 17], and an indirect neuroprotective effect via blockade of the inducible nitric oxide synthase (iNOS), resulting in the reduction of secondary tissue damage [18].

OPN acts as a potent chemoattractant. It therefore promotes migration of mesenchymal stem cells [19], endothelial cells [20], macrophages [21], and hematopoietic stem cells [22]. Within the central nervous system (CNS), Yan et al. were the first to associate a lack of OPN after intracerebral hemorrhage or ischemia with a significant decrease in neuroblast migration [23, 24]. We recently reported that OPN supports not only the migration of neural stem cells (NSC) in monolayer culture, but also their survival under stressful conditions, their proliferation, and their differentiation into neurons. These effects were in part mediated via the CXC chemokine receptor type 4 (CXCR4), thus unraveling a new molecular mechanism of the putative action of OPN on NSC [25]. Based on these in-vitro data, we hypothesized that OPN would possess similar effects in vivo, particularly in the context of cerebral ischemia.

The DCX-luc transgenic mouse expresses the firefly luciferase (luc) under the control of the doublecortin (DCX) promoter, allowing for the noninvasive visualization of DCX-positive neuroblasts in the brain in vivo using bioluminescence imaging (BLI) [26]. Thus, we here aimed to establish and visualize the effects of OPN in a longitudinal study under physiological conditions as well as after cerebral ischemia in vivo using BLI.

## Methods

### Animals and surgery

All animal procedures followed the German laws for animal protection and were approved by the local animal care committee as well as local governmental authorities (Landesamt für Natur, Umwelt und Verbraucherschutz North Rhine-Westphalia, LANUV). Results are reported in compliance with the ARRIVE guidelines (Animal Research: Reporting In Vivo Experiments). To exclude putative influences of hormonal changes on the findings, we used only male mice. For surgery (photothrombosis (PT) or injections of osteopontin or phosphate-buffered saline (PBS), respectively), 46 DCX-luc transgenic mice expressing a firefly luciferase in DCX^+^ cells with C57/Bl6 albino background (B6(Cg)-Tyrc-2 J/J; Paracelsus Medical University, Salzburg, Austria), aged 7–9 weeks old, weighing 20–25 g, were anesthetized with 4% isoflurane via a face mask as well as local, subcutaneous bupivacaine hydrochloride injections. Anesthesia was maintained with 2–4% isoflurane in a 65%/35% nitrous oxide/oxygen atmosphere. Throughout the surgical procedures, a thermostatically controlled heating pad maintained the body temperature at 37.0 °C. After surgery, the mice were transferred to a heated recovery box to recover from anesthesia. Mice were put back into their individually ventilated home cages under 12 h light/12 h darkness cycle, and water and food were provided ad libitum*.*

#### Photothrombosis

In one group of DCX-luc transgenic mice (*n* = 27), focal cerebral ischemia was induced by PT as described previously [27]. In brief, animals were anesthetized, placed in a stereotactic frame, and an approximately 3-mm long, median incision of the skin between the caudal ends of the ears was made. The periost was removed, and bregma and lambda points were identified. Protected from light, the photosensitive dye Rose bengal (Sigma Aldrich, St. Louis, USA) was dissolved in sterile 1% PBS at a concentration of 10 mg/ml, and 0.1 ml was injected intraperitoneally 5 min before illumination. A fiberoptic bundle of an LED light source (Zeiss CL6000 LED, Carl Zeiss, Oberkochen, Germany) with a 50-mm aperture was centered using a micromanipulator at 1.8 mm laterally and 2.5 mm posterior from the bregma as close as possible to the skull. The mouse brain was illuminated through the intact skull for 15 min starting 5 min after intraperitoneal injection of Rose bengal. Afterwards, the skin was closed with sutures and the animals were transferred to a heated recovery box before moving them back to the home cage; 19 control mice did not undergo this PT procedure.

#### OPN treatment

Animals were treated with a single intracerebroventricular (i.c.v.) injection of 0.6 μg OPN (recombinant mouse, R&D Systems, Minneapolis, USA) in 3 μl sterile 1% PBS to allow the assessment of the effects of OPN on the migration of neuroblasts in vivo. OPN treatment was performed in eight healthy (naive) mice, and in 14 stroke mice on the day after induction of PT. The respective control groups received an i.c.v. injection of 3 μl sterile 1% PBS (vehicle) alone (naive n = 8; stroke *n* = 13), with assignment to the treatment or control group being performed by randomization. Another control group of three mice was subjected only to the skull damage associated with treatment without the i.c.v. injection itself, by simply drilling a borehole over the lateral ventricle (for an overview of the experimental groups, see Table 1). To localize the lateral ventricle in individual mice, magnetic resonance imaging (MRI) data were used for a precise localization, as described below. Based on MRI data, the i.c.v. injection was performed using a micromanipulator at the following coordinate ranges from the bregma: 1.2–1.4 mm ML, 0.2–0.4 mm AP, 2.6–3.0 VD.

**Table 1:** Overview of the experimental groups

	Control		OPN
Group	Only borehole	ICV injection PBS (vehicle)	ICV injection OPN
Healthy (naive)	*n* = 3	*n* = 8	*n* = 8
Stroke	–	*n* = 13	*n* = 14

*ICV* intracerebroventricular, *OPN* osteopontin, *PBS* phosphate-buffered saline

#### Labeling of proliferating cells

To label proliferating cells in vivo, the tracer bromodeoxyuridine (BrdU) was injected intraperitoneally into selected animals (naive *n* = 8; stroke *n* = 12). Injections were started immediately after i.c.v. injections of OPN or vehicle, respectively, and repeated twice, after 12 and 24 h. BrdU was injected at a dose of 50 mg/kg body weight per injection, resulting in a cumulative dose of 150 mg/kg body weight BrdU per animal.

### MRI protocol

In all mice, MRI was performed directly before i.c.v. injection of OPN or vehicle, respectively, to localize the lateral ventricles precisely and to characterize the extent of the ischemic lesion in animals with photothrombotic stroke. Animals were anesthetized with 4% isoflurane. Anesthesia was maintained with 2–4% isoflurane in a 65%/35% nitrous oxide/oxygen atmosphere. To monitor physiological parameters during MRI, an MR compatible monitoring system (Small Animal Instruments Inc., NY, USA) was employed and connected to a custom-made data acquisition program based on DASYLab (measX, Mönchengladbach, Germany). A fiberoptic temperature probe and a respiration pad were used to collect data concerning body temperature and respiratory rate as well as their corresponding waveforms. A thermostatically controlled water flow system connected to a heating pad was employed to maintain body temperature at 37 ± 0.5 °C. All experiments were conducted on a 11.7-Tesla BioSpec system (Bruker, BioSpin, Ettlingen, Germany) with a BGA9s gradient system with a maximum gradient 750 mT/m and a minimum rise time of < 250 μs. The transmission was achieved with a quadrature volume resonator (inner diameter 72 mm). A standard rat brain quadrature surface coil (~ 30 × 30 mm^2^ Bruker, BioSpin, Ettlingen, Germany) detected the signal. The animals were positioned prone in a dedicated cradle with a stereotactic head holder and with the surface coil placed directly over their head. Twelve contiguous 1-mm thick T2-weighted coronal images of the brain were acquired (TR/TE = 3000/14 ms, 16 echoes, FOV = 3.0 cm, matrix 128 × 128), using a multislice-multiecho Carr-Purcell-Meiboom-Gill (CPMG) sequence.

### BLI protocol

BLI was performed on the day before induction of photothrombosis, 2 days before i.c.v. injection of naive animals, as well as 2, 7, 10, 14, 21, and 28 days after OPN or control treatment. For BLI, DCX-luc transgenic mice were anesthetized with 5% isoflurane in a 65%/35% nitrous oxide/oxygen atmosphere and shaved on the head as well as in the neck region. Animals were intraperitoneally injected with luciferin at 300 mg/kg body weight (d-luciferin in potassium salt 99%, Synchem, Altenburg, Germany), leading to its oxidization by luciferase active under the DCX promoter, therefore causing light emission from neuroblasts. One to three minutes after luciferin injection, images were acquired every minute for 30 min in total, using an IVIS Spectrum CT system (Perkin Elmer, Massachusetts, USA), measuring average radiance of photons/second/cm^2^/steradian (p/s/cm^2^/sr), or total flux in photons/second, respectively (p/s). During the 30 min of acquisition, animals were placed on a 37 °C controlled heating pad with isoflurane levels between 2 and 4%.

### Image analyses

#### MRI

MRI data from animals with ischemic lesions were analyzed using the software Vinci (Max-Planck-Institute for Metabolism Research, Cologne, Germany). Adjusted ellipsoid volumes of interest (VOIs) were placed to cover the infarct zone and to measure the infarct volume in mm^3^ 1 day after PT.

#### BLI

An investigator blinded to the treatment group (RR) analyzed the BLI data using the Living Image 4.4 (Caliper Life Sciences, Massachusetts, USA). Size-constant regions of interest (ROIs) were placed on the ipsi- (right; infarct lesion) and contralesional (left) hemisphere of the head measuring total flux in p/s. To eliminate intra- and interindividual variations of total flux, the ratio between the right and left hemisphere was calculated for each animal individually. To analyze the migration of the hotspot of DCX-labeled cells, BLI images of each time point and animal with the maximum of total flux were identified using Living Image 4.4 (Caliper Life Sciences, Massachusetts, USA). For each animal and each time point, an individualized scale was set to visualize the hotspot. Using the software ImageJ (National Institutes of Health, Bethesda, USA), the respective hotspot was encircled, and the center of the circle was marked. To determine any migration of the hotspot, the distance from the middle of the circle to the midline was measured in mm.

### Immunohistochemistry

For immunohistochemical workup at different time points, a few animals were sacrificed at earlier time points (*n* = 2 naive mice each after 2 and 7 days; *n* = 2 stroke mice each after 2, 7, and 14 days). All remaining animals were sacrificed 1 day after the last BLI session on day 28. Mice were deeply anesthetized with ketamine (0.1 mg/kg) and xylazine (0.01 mg/kg) and perfused with a fixation solution of 4% paraformaldehyde. The brains were rapidly removed, frozen in 2-methylbutane, and stored at −80 °C before histological and immunohistochemical processing. Coronal brain sections (slice thickness 10 μm) were cut and stained with polyclonal anti-doublecortin (DCX) to label neuroblasts and young neurons (dilution 1:800, Santa Cruz Biotechnology Inc., Santa Cruz, USA, cat. no. sc-8066; or dilution 1:1.500, Abcam, Cambridge, United Kingdom, cat. no. ab18723) and monoclonal rat anti-BRDU to label proliferating cells (dilution 1:200, Abcam, Cambridge, United Kingdom, cat. no. ab6326). For BRDU staining, DNA hydrolysis was performed by incubation in 1 M HCL for 10 min at 37 °C. Sections were counterstained with Hoechst to label all nuclei (Hoechst 33,342, Thermo Fisher Scientific, Massachusetts, USA). A fluorescent-labeled secondary antibody was used for visualization (dilution 1:500, Alexa-Fluor −488 and −568, Invitrogen, Thermo Fisher Scientific, Waltham, Massachusetts, USA). Additionally, the blood-brain barrier (BBB) disruption was visualized by incubation with a biotinylated anti-mouse IgG secondary antibody alone (dilution 1:100, Vector Laboratories, Burlingame, California, USA). For visualization, the ABC Elite kit (Vector Laboratories, Burlingame, USA), with diaminobenzidine (Sigma-Aldrich, Munich, Germany) as the final reaction product, was used.

Representative pictures were taken with an inverted fluorescence phase-contrast microscope (Keyence BZ-9000E, Keyence, Osaka, Japan). The size of the subventricular zone (SVZ) as the major neurogenic niche was determined for each animal by measuring the area covered by DCX-positive cells, as described previously [28].

### Statistical analyses

Descriptive statistics and Student’s *t* tests were performed with Microsoft Excel 2010 (Microsoft Corp.). For comparison of multiple groups, one-way analysis of variance (ANOVA) followed by Tukey-HSD as a post-hoc test for data that conformed to the assumption of homogeneity of variance, and Games-Howell for data that did not fit to the assumption of homogeneity of variance, was performed with IBM SPSS Statistics 24.0 for Windows (International Business Machines Corporation IBM, Armonk, New York, USA). Statistical significance was set at less than 5% level (*p* < 0.05). One-way ANOVA results were stated as the F-ratio: F (degrees of freedom of the between group, degrees of freedom of the within group) = F-ratio.

## Results

### OPN enhances the directed migration of neuroblasts

**Fig. 1:**
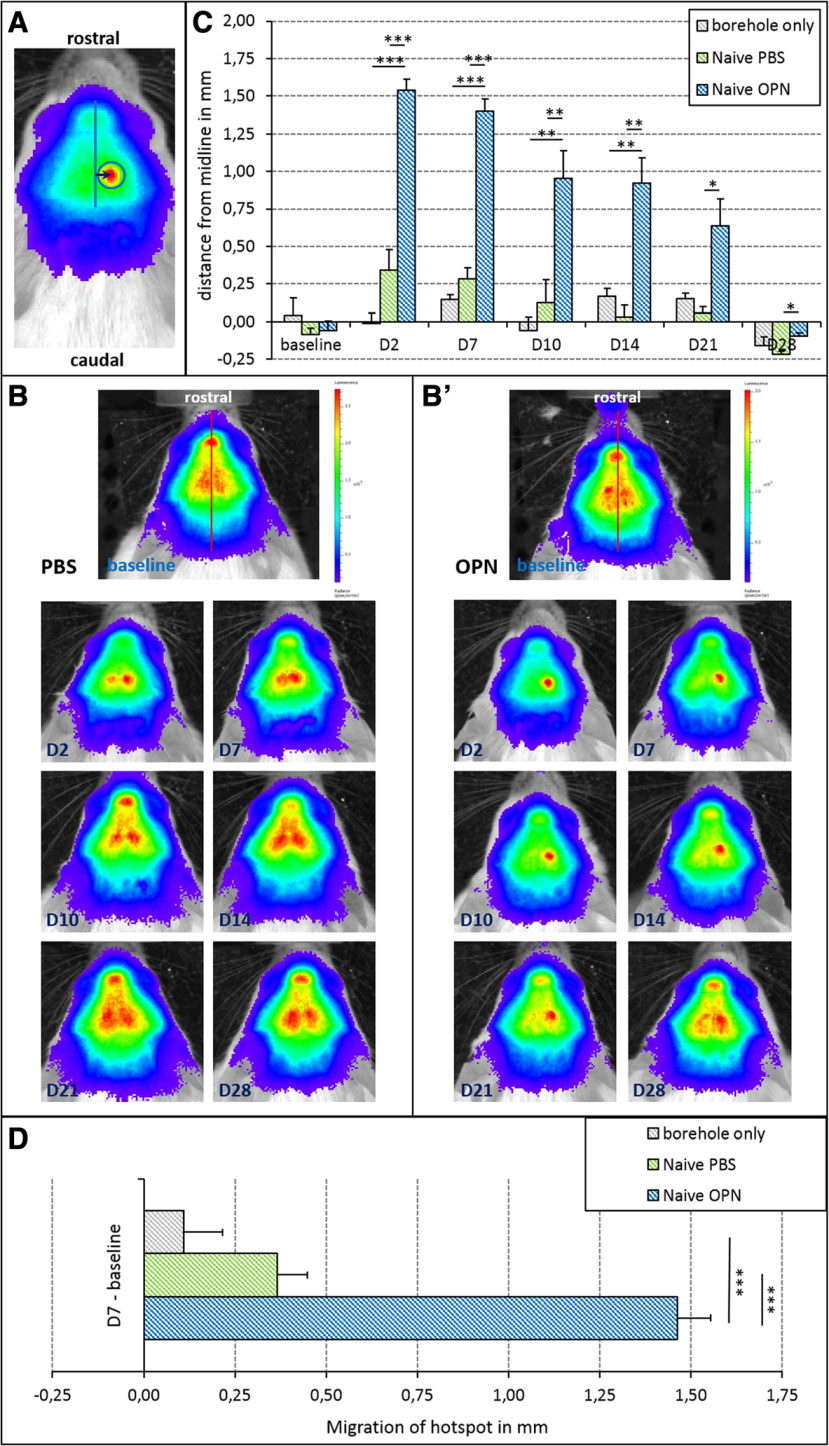
Osteopontin (OPN) enhances migration of neuroblasts in the healthy (naive) brain. OPN was applied at 0.6 μl in phosphate-buffered saline 1% (PBS) via a single intracerebroventricular (i.c.v.) injection into the brains of healthy mice; injection of PBS alone as vehicle served as control. A second control group only received a borehole to the skull without i.c.v. injection. **a** Representative image of a mouse head with doublecortin-positive (DCX^+^) neuroblasts visualized as a ‘hotspot’ (red) using bioluminescence imaging (BLI). The arrow marks the distance between the midline and the maximal BLI signal (hotspot), thereby measuring the length of lateral migration of neuroblasts. **b** Representative images of neuroblast migration in a healthy PBS-injected (left) and a healthy OPN-injected mouse (right) before (baseline) and at various time points after treatment. In the OPN-treated animal, the ‘hotspot’ of maximal BLI signal lateralized to the right hemisphere between day (D)2 and D14, while no lateralization was observed in the control mouse. Note that for better visualization of the location of the ‘hotspots’, different scales were used for the left and right panels. **c** Quantification of the distance of migration revealed that at baseline (i.e., before any treatment) the maximal BLI signal was localized very close to the midline. Between D2 and D21 after injection of OPN into the right lateral ventricle, the distance of the maximal BLI signal to the midline was significantly increased, indicating migration of neuroblasts towards the injection site of OPN. Both control groups (PBS injection and borehole only) did not display significant movement of the BLI signal towards the manipulated hemisphere (means ± SEM; *p < 0.05; **p < 0.01; ***p < 0.001). **d** To judge the absolute distance of neuroblast migration, the distance between the baseline signal and the signal 7 days after treatment was measured for each group of differently treated mice. The distance covered by neuroblasts in OPN-injected mice was significantly greater than that in PBS-injected animals or those with a borehole only (means ± SEM; ***p < 0.001)

DCX-luc transgenic mice were treated with a single injection of OPN into the right lateral ventricle for the assessment of the effect of OPN on DCX^+^ neuroblasts in the healthy (naive) brain. Control groups received either an i.c.v. injection of vehicle (PBS) or a borehole over the right lateral ventricle only (see Table 1). The treatment effect on neuroblast migration towards the site of injection/manipulation was visualized via BLI, measuring the distance from the midline to the ‘hotspot’ of maximal BLI signal (Fig. 1a). Baseline imaging data acquired before treatment depicted the maximal BLI signal close to the midline in all three groups (*p* = 0.942; Fig. 1b). Between days 2 and 21 after injection of OPN into the right lateral ventricle, the BLI signal was observed at significantly greater distance to the midline than under control conditions, suggesting that OPN directly triggers migration of neuroblasts (OPN vs. PBS: day 2 F(2,27) = 51.87, *p* < 0.01; day 7 F(2,27) = 83.11, *p* < 0.001; day 10 F(2,27) = 9.82, *p* < 0.01; day 14 F(2,27) = 15.45, *p* < 0.01; day 21 F(2,27) = 6.6, *p* < 0.05; OPN vs. borehole: day 2 F(2,27) = 51.87, *p* < 0.01; day 7 F(2,27) = 83.11, *p* < 0.001; day 10 F(2,27) = 9.82, *p* < 0.01; day 21 F(2,27) = 15.45, *p* < 0.01; Fig. 1b, c). After 28 days, maximal BLI signal was observed in the contralateral hemisphere for all three groups of mice, with the hotspot closest to the midline in OPN animals (F(2,27) = 5.03, *p* < 0.05; Fig. 1c). Between the two control groups (PBS injection vs. borehole only) there was no significant difference over the period of 28 days (day 2, *p* = 0.128; day 7, *p* = 0.527; day 10, *p* = 0.785; day 14, *p* = 0.795; day 21, *p* = 0.892; day, 28 *p* = 0.441), suggesting that the effect seen with OPN was specific and not due to any type of manipulation (Fig. 1b, c). To assess the absolute distance of neuroblast migration, the distance between the baseline signal and the maximal signal 7 days after treatment was measured for each group of differently treated mice. The distance covered by neuroblasts in OPN-injected mice was significantly longer than that in either control group (F(2,27) = 59.97, *p* < 0.001; Fig. 1d).

### OPN expands neuroblast numbers

**Fig. 2:**
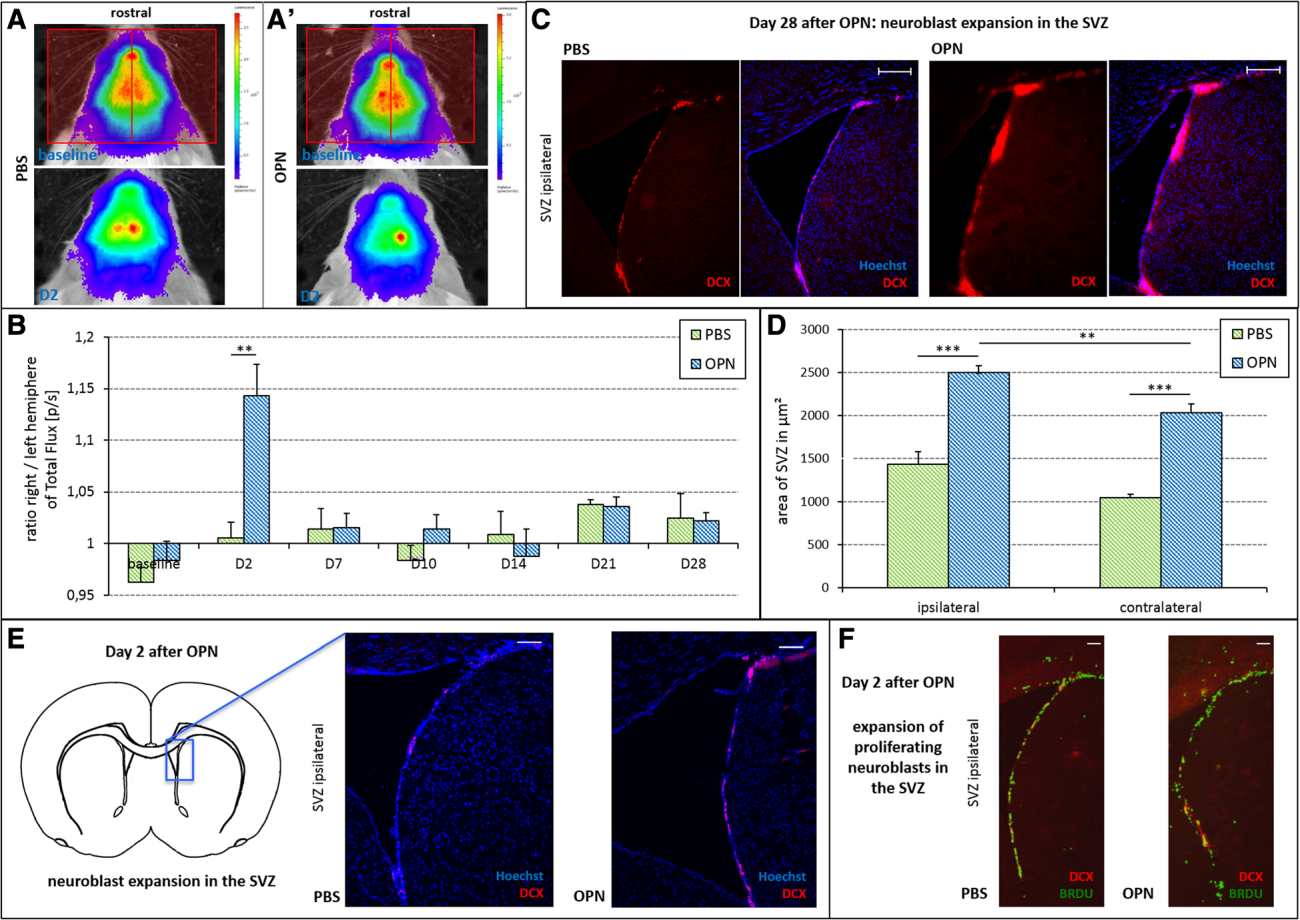
Osteopontin (OPN) expands the number of neuroblasts in the healthy brain. Before (baseline) and at various time points after treatment, the total flux of photons was measured via bioluminescence imaging (BLI) as a surrogate for the number of doublecortin-positive (DCX^+^) neuroblasts. **a** Quantification of total flux was performed for each hemisphere and time point, as demonstrated on representative images, and a ratio was calculated between the hemispheres (the red rectangles in the baseline scan demonstrate measured ROIs of ipsi- and contralateral hemisphere). **b** Two days (D2) after i.c.v. injection of OPN, the ratio between right (injected) and left (contralateral) hemisphere regarding the total flux of photons was significantly higher compared with phosphate-buffered saline (PBS)-injected control mice, suggesting an expansion of DCX^+^ neuroblasts (means ± SEM; **p < 0.01). **c** Representative immunohistochemical images of DCX^+^ cells (red) costained with a nuclear marker (Hoechst; blue) in the subventricular zone (SVZ) ipsilateral to the injection. The number of DCX^+^ cells was increased 28 days after OPN treatment (right panels) compared with control (left panels; scale bars = 200 μm). **d** Twenty-eight days after injection of OPN, the area covered by DCX^+^ neuroblasts was significantly increased. This effect was more pronounced in the SVZ ipsilateral to injection compared with the contralateral side (means ± SEM; **p < 0.01, ***p < 0.001). **e** Representative immunohistochemical images of DCX^+^ cells (red) costained with a nuclear marker (Hoechst; blue) in the SVZ ipsilateral to the injection. The number of DCX^+^ cells was increased 2 days after OPN treatment (right panel) compared with control (left panel; scale bars = 100 μm). **f** Representative immunohistochemical images costaining bromodeoxyuridine (BrdU; green; labeling proliferating cells) and DCX (red; labeling neuroblasts) in the SVZ ipsilateral to the injection. The number of costained proliferating neuroblasts (BrdU^+^/DCX^+^) was increased 2 days after OPN treatment (right panel) compared with control (left panel; scale bars = 100 μm)

To assess the effect of OPN on the extent of neurogenesis, BLI was used to quantify the emission of photons emanating from DCX^+^ neuroblasts in DCX-luc mice, measured as total flux (p/s). Data are expressed as a ratio between the right (injected) and the left (contralateral) hemisphere (Fig. 2a). Two days after injection of OPN into the right lateral ventricle, the total flux ratio was already increased in OPN-treated mice compared with PBS-injected controls, suggesting an expansion of DCX^+^ neuroblasts in the ipsilateral hemisphere (F(1,22) = 16.28, *p* < 0.01; Fig. 2a, b). Ex vivo, DCX^+^ neuroblasts were immunohistochemically identified in the SVZ as one of the main neurogenic stem cell niches in the mammalian brain (Fig. 2c). Injection of OPN into the lateral ventricle significantly induced an expansion of neuroblasts in the SVZ 28 days after OPN treatment (F(3,86) = 28.7; *p* < 0.001). This effect was more pronounced in the SVZ ipsilateral to injection compared with the contralateral side (*p* < 0.01; Fig. 2d). Likewise, a similar result of an OPN-induced expansion of neuroblasts in the SVZ was already visible 2 days after OPN treatment (Fig. 2e). Moreover, OPN increased the numbers of BrdU^+^/DCX^+^ proliferating neuroblasts in the SVZ 2 days after OPN treatment (Fig. 2f).

### OPN induces neuroblast migration after focal cortical ischemia

**Fig. 3:**
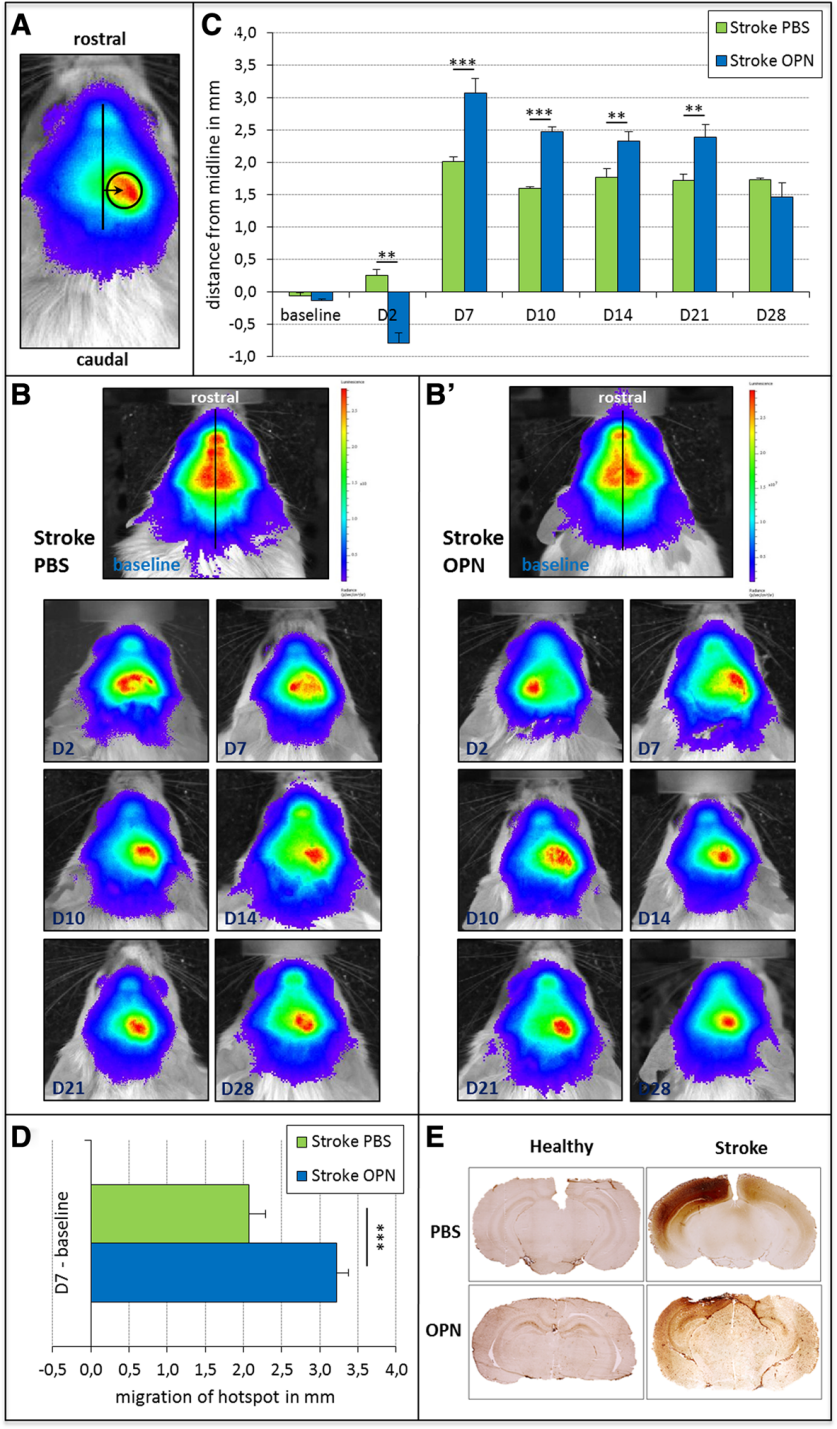
Osteopontin (OPN) induces neuroblast migration after stroke. One day after photothrombosis (PT), 0.6 μg OPN was injected into right lateral ventricle. Control groups received an injection of vehicle (phosphate-buffered saline (PBS)) after PT. **a** Representative image of a mouse head with the ‘hotspot’ of maximal bioluminescence imaging (BLI) signal over the right (i.e., lesioned) hemisphere. The arrow marks the distance between the midline and the ‘hotspot’, thereby measuring the distance of neuroblast migration from the midline. **b** Representative images of neuroblast migration in stroke mice after injection of PBS (left panels) or OPN (right panels) before (baseline) and at various time points after treatment. Photothrombotic stroke qualitatively elicited a migration of neuroblasts towards the lesioned hemisphere, both after OPN treatment and in controls. Note that for better visualization of the location of the ‘hotspots’, different scales were used for the left and right panels. **c** Before photothrombotic stroke, the maximal BLI signal was located close to the midline in all animals (baseline). After ischemic stroke, neuroblasts migrated towards the lesioned hemisphere in all groups of mice. In this, neuroblasts covered a greater distance in OPN-treated animals between day (D)7 and D21 after treatment compared with the controls. Note that 2 days after injection, the maximal BLI signal was localized in the contralateral hemisphere in OPN-treated mice (means ± SEM; **p < 0.01; ***p < 0.001). **d** The absolute distance of neuroblast migration was measured as the distance between the baseline signal and the signal 7 days after treatment for each group of mice. The distance covered by neuroblasts in OPN-injected mice was significantly greater than that in the control group (means ± SEM; ***p < 0.001). **e** Representative immunohistochemical images of blood-brain barrier (BBB) disruption 2 days after focal cerebral ischemia. Healthy animals (left panels) did not show any signs of BBB disruption, whereas stroke animals (right panels) suffered from a more widespread BBB disruption around the peri-infarct area irrespective of control (upper right panel) or OPN treatment (lower right panel)

The mice were subjected to photothrombosis (PT) as a model of focal cortical ischemia to assess the effects of OPN on the migration and generation of neuroblasts after ischemic stroke. MRI 1 day after stroke verified the ischemic lesions and ensured that only mice with homogenous lesions of approximately equal size were randomized into the different treatment groups (average lesion size 27.3 ± 5.1 mm^3^ in OPN-treated mice, and 27.4 ± 4.2 mm^3^ in control animals, *p* = 0.989). OPN or PBS (as the vehicle for control) were then injected into the lateral ventricle ipsilateral to ischemia. Similarly to the nonischemic mice, migration of neuroblasts was quantified as the distance of the ‘hotspot’ of maximal BLI signal from the midline (Fig. 3a, see Fig. 1a). After photothrombotic stroke, a lateralization of DCX^+^ neuroblasts towards the ischemic hemisphere was observed during the entire period of observation, both after OPN treatment as well as after PBS injection (Fig. 3b, c). However, injection of OPN significantly enhanced this neuroblast migration between days 7 and 21 following treatment (day 7 F(1,40) = 14.67, *p* < 0.001; day 10 F(1,40) = 21.15, *p* < 0.001; day 14 F(1,40) = 8.35, *p* < 0.01; day 21 F(1,40) = 10.91, *p* < 0.01; Fig. 3c). Of note, 2 days after injection, the maximal BLI signal was localized in the contralateral hemisphere in OPN-treated mice only, potentially suggesting increased recruitment of stem cells from the contralateral SVZ (F(1,40) = 3.11, *p* < 0.01; Fig. 3b, c). The absolute distance covered by neuroblasts exposed to an excess of OPN by day 7 after injection was increased compared with control conditions (F(1,40) = 17.13, *p* < 0.001; Fig. 3d). Intriguingly, the distance of stem cell migration observed after ischemia roughly doubled the distance seen in the experimental set of naive mice (see *y* axis in Figs. 1c and 3c). To characterize disruption of the BBB after stroke, tissue was incubated with a biotinylated anti-mouse IgG secondary antibody alone, impressively visualizing ischemia-induced BBB damage (Fig. 3e). However, the extent of BBB disruption remained unaltered by OPN treatment.

### OPN supports neurogenesis after focal cortical ischemia

**Fig. 4:**
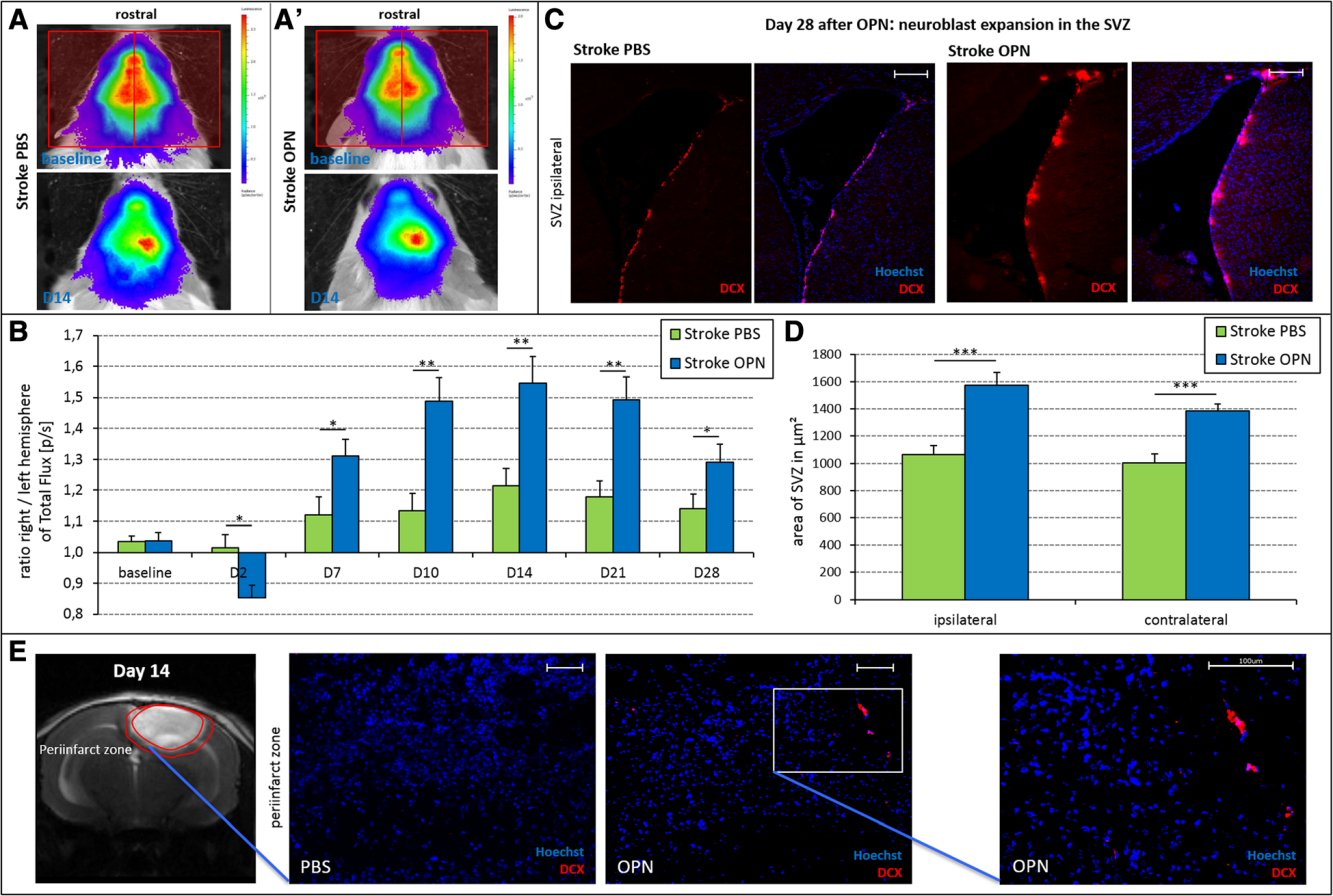
Osteopontin (OPN) supports neurogenesis after stroke. Before photothrombosis (PT) (baseline) and at various time points after treatment, the total flux of photons was measured via bioluminescence imaging (BLI) as a surrogate for the number of doublecortin-positive (DCX^+^) neuroblasts. **a** Quantification of total flux was performed for each hemisphere and time point, as demonstrated on representative images, and a ratio was calculated between the hemispheres (the red rectangles in the baseline scan demonstrate measured ROIs of ipsi- and contralateral hemisphere). **b** Between day (D)7 and D28 after i.c.v. injection of OPN into stroke mice, the ratio between right (injected) and left (contralateral) hemisphere regarding the total flux of photons was significantly higher compared to phosphate-buffered saline (PBS)-injected stroke mice, consistent with increased expansion of DCX^+^ neuroblasts (means ± SEM; **p < 0.01). Note that 2 days after injection, total flux of photons was transiently enhanced in the contralateral hemisphere of OPN-treated mice (means ± SEM; *p < 0.05; **p < 0.01). **c** Representative immunohistochemical images of DCX^+^ cells (red) costained with Hoechst (blue) in the subventricular zone (SVZ) ipsilateral to the i.c.v. injection in mice subjected to photothrombotic stroke. The number of DCX^+^ cells was increased 28 days after OPN treatment (right panels) compared with control (left panels; scale bars = 200 μm). **d** In mice subjected to photothrombotic stroke, the area covered by DCX^+^ cells in the SVZ was significantly increased in OPN-treated mice compared with PBS-injected controls after 28 days. This was observed in both SVZ, ipsi- as well as contralateral to i.c.v. injection (means ± SEM; ***p < 0.001). **e** Representative immunohistochemical images reveal DCX^+^ neuroblasts (red) costained with Hoechst (blue) in the peri-infarct area in mice 14 days after OPN treatment (right panels) but not in control animals (left panels; scale bars = 100 μm)

To assess the effects of OPN on neurogenesis in the context of photothrombotic stroke, photon emission emanating from DCX^+^ neuroblasts was quantified and expressed as a ratio between lesioned/injected and the unlesioned (contralateral) hemisphere (Fig. 4a). In OPN-injected mice, this ratio was higher between days 7 and 28 after treatment, suggesting an enhanced expansion of neuroblasts (day 7 F(1,40) = 5.92, *p* < 0.05; day 10 F(1,40) = 13.94, *p* < 0.01; day 14 F(1,40) = 10.53, *p* < 0.01; day 21 F(1,40) = 12.20 *p* < 0.01; day 28 F(1,40) = 4.13, *p* < 0.05; Fig. 4a, b). Again, 2 days after injection, total flux of photons was transiently increased in the contralateral hemisphere of OPN-treated mice (F(1,40) = 7.17, *p* < 0.05), potentially suggesting enhanced recruitment of neuroblasts from the contralateral SVZ (Fig. 4b). Comparing the magnitude of effects seen after ischemia and in naive mice, ischemia robustly increased the extent of neuroblast expansion, as revealed by total flux (see Figs. 2b and 4b).

Immunohistochemical staining revealed an increased number of DCX^+^ cells 28 days after OPN treatment compared with the control, thereby corroborating the in-vivo data (Fig. 4c). The area of the SVZ covered by DCX^+^ neuroblasts was significantly enlarged following OPN treatment, both in the ipsilateral as well as the contralateral hemisphere (F(3,86) = 14.05, *p* < 0.001; Fig. 4d). To detect neuroblasts migrating towards the ischemic lesion, DCX stainings were analyzed in the peri-infarct region 14 days after stroke. Interestingly, NSC^+^ neuroblasts were detected in the peri-infarct region only after OPN treatment, but not after PBS injection **(**Fig. 4e**)**.

## Discussion

We investigated the effect of OPN on neuroblasts in healthy mice as well as after focal cerebral ischemia. We observed that OPN supports directed migration as well as an expansion of the stem cell niche in vivo, as assessed by bioluminescence imaging (BLI).

Despite significant advances in regenerative medicine, the noninvasive visualization and quantification of endogenous neural progenitor cells in vivo remains challenging [29]. One current approach to visualize neural progenitors in vivo is the use of transgenic mice expressing the bioluminescent protein firefly luciferase under the control of the DCX promoter that is characteristic for neural progenitors, thus rendering the endogenous progenitor niche detectable by BLI [26]. In these mice, emission of photons emanating from DCX^+^ neuroblasts is quantified and reflects the neuroblast numbers; i.e., a quantitative increase in BLI signal indicates neurogenesis [26, 30]. We here established an additional read-out parameter in the DCX-luc mouse: the distance of the maximal BLI signal from the midline. Since at baseline this maximal signal, or ‘hotspot’, is located exactly in the midline, its distance from the midline indicates the migration of DCX^+^ neuroblasts. We chose those two distinct measures in analogy to our previous work in vitro where OPN independently affected proliferation and migration of primary NSC [25].

One potential pitfall reported with the DCX-luc model is that the disruption of the BBB may result in an increased BLI signal [31]. Since focal cerebral ischemia indeed disrupts the BBB, we included a control group of animals subjected to ischemia alone. Immunohistochemically, no relevant difference in the extent of BBB disruption between the verum and placebo group was found. Two days after OPN treatment, the maximal BLI signal was found in the contralateral hemisphere in OPN-treated mice only. One interpretation of this phenomenon could be increased recruitment of stem cells from the contralateral SVZ. Alternatively, a midline shift—evoked by peri-infarct edema—in addition to an enhanced BLI signal due to BBB disruption could also misdirect the BLI signal to the contralateral side. However, this would have been expected in both the verum and placebo group. Moreover, we confirmed the effects of OPN on neurogenesis by immunohistochemistry.

Overcoming such potential methodological drawback with BLI, we previously visualized activation and recruitment of NSC in the rat using an innovative positron emission tomography (PET) technique [32]. The present study corroborates and extends these previous findings, using an alternative visualization technique in mice. Thus, although BBB disruption may interfere with a direct comparison of effects, stem cell migration after cerebral ischemia was increased two-fold compared with that in naive mice (see Figs. 2 and 4). As a conclusion, and with the utmost caution, data suggest that osteopontin and the ischemic lesion may act synergistically in mobilizing stem cells.

The DCX-luc mouse has recently been used to visualize endogenous neurogenesis after experimental stroke induced by transiently occluding the middle cerebral artery, a stroke model that leads to more extensive ischemic lesions than the ones induced by photothrombosis as used in our study [30]. Interestingly, Adamczak et al. reported that while old animals display less basal neurogenesis than young mice, they respond to ischemic stroke with enhanced sensitivity and even recruit additional neuroblasts from the contralateral hemisphere, resulting in neurogenesis levels similar to young animals [30]. In line with this report, we observed recruitment of neural progenitors from the hemisphere contralateral to the ischemic stroke induced by OPN. Since upregulation of neurogenesis after ischemic stroke does not seem to decline with age, we suggest that mammals of any age would benefit from a pharmacological treatment aimed at enhancing progenitor proliferation.

Other groups have used bioluminescence imaging to monitor neural progenitor cells by transfecting them with the *luciferase* gene in vivo using viral vectors [33–35]. However, this approach labels only a subset of neural progenitors in the SVZ, while our experimental setup was designed to visualize the entire progenitor population expressing doublecortin. Using lentiviral vector-mediated labeling and BLI, Vandeputte et al. analyzed stem cell proliferation after photothrombotic stroke. Corresponding to our results, they reported an increased BLI signal between 2 days and 2 weeks after a stroke that decreased after 3 months [35]. Using the same stroke model, we here observed that OPN can augment upregulation of progenitor proliferation.

Neurogenesis is a central aspect of regenerative medicine. Various neurological disorders associated with neuronal loss are accompanied by concomitant upregulation of endogenous neurogenesis, as is the case after cerebral hypoxia [36], focal cerebral ischemia [37, 38], or in neurodegenerative diseases such as Alzheimer’s [39] and Huntington’s disease [40]. However, in most cases, this physiological upregulation of neurogenesis is insufficient to enable functional recovery. Multiple approaches have been taken to stimulate neurogenesis in the adult brain, including pharmacological means [41–44], as well as nonpharmacological approaches such as transcranial direct current stimulation [45–47].

## Conclusion

We recently reported that OPN induces neurogenesis from neural stem cells in vitro, alongside the promotion of stem cell survival, proliferation, and migration, mediated by the CXC chemokine receptor type 4 (CXCR4) [25]. The current study extends these data in an in-vivo stroke model and corroborates earlier results by Yan et al. showing that absence of OPN impairs neuroblast migration towards an ischemic or hemorrhagic brain lesion [23, 24]. Besides proliferation and migration of neural progenitors, neuroinflammatory processes including hematogenous as well as brain-resident immune cells characterize the days and weeks after stroke [48–51]. This immune response includes an extensive upregulation of OPN in microglia and macrophages, where it serves as an essential regulatory protein [11, 12, 14, 15]. We recently reported that OPN modulates microglia function by shifting their inflammatory profile towards an anti-inflammatory phenotype [52]. Thus, OPN may be considered a dual role player after stroke, recruiting neural progenitors and harnessing neuroinflammation at the same time.

## Abbreviations

ANOVA: Analysis of variance
BBB: Blood-brain barrier
BLI: Bioluminescence imaging
BrdU: Bromodeoxyuridine
CNS: Central nervous system
DCX: Doublecortin
i.c.v.: Intracerebroventricular
iNOS: Inducible nitric oxide synthase
luc: Luciferase
MRI: Magnetic resonance imaging
NSC: Neural stem cells
OPN: Osteopontin
PBS: Phosphate-buffered saline
PET: Positron emission tomography
PT: Photothrombosis
ROI: Region of interest
SVZ: Subventricular zone
VOI: Volume of interest
